# Noradrenergic Enhancement of Motor Learning, Attention, and Working Memory in Humans

**DOI:** 10.1093/ijnp/pyab006

**Published:** 2021-02-22

**Authors:** Hsiao-I Kuo, Feng-Xue Qi, Walter Paulus, Min-Fang Kuo, Michael A Nitsche

**Affiliations:** 1 School and Graduate Institute of Physical Therapy, College of Medicine, National Taiwan University, Taipei, Taiwan; 2 Key Laboratory of Sport Training of General Admission of Sport of China, Beijing Sport University, Xinxin Road, Haidian District, Beijing, China; 3 Department of Sport Training, Sport Coaching College, Beijing Sport University, Beijing, China; 4 Department of Clinical Neurophysiology, University Medical Center, Georg-August-University, Göttingen, Germany; 5 Dept. Psychology and Neurosciences, Leibniz Research Centre for Working Environment and Human Factors, Dortmund, Germany; 6 Department of Neurology, University Medical Hospital Bergmannsheil, Bochum, Germany

**Keywords:** Attention, memory, motor learning, noradrenaline, reboxetine

## Abstract

**Background:**

Noradrenaline has an important role as a neuromodulator of the central nervous system. Noradrenergic enhancement was recently shown to enhance glutamate-dependent cortical facilitation and long term potentiation-like plasticity. As cortical excitability and plasticity are closely linked to various cognitive processes, here we aimed to explore whether these alterations are associated with respective cognitive performance changes. Specifically, we assessed the impact of noradrenergic enhancement on motor learning (serial reaction time task), attentional processes (Stroop interference task), and working memory performance (n-back letter task).

**Methods:**

The study was conducted in a cross-over design. Twenty-five healthy humans performed the respective cognitive tasks after a single dose of the noradrenaline reuptake inhibitor reboxetine or placebo administration.

**Results:**

The results show that motor learning, attentional processes, and working memory performance in healthy participants were improved by reboxetine application compared with placebo.

**Conclusions:**

The results of the present study thus suggest that noradrenergic enhancement can improve memory formation and executive functions in healthy humans. The respective changes are in line with related effects of noradrenaline on cortical excitability and plasticity.

**Significance Statement**

Acute enhancement of noradrenergic activation via a single dose of reboxetine (RBX) can improve implicit motor learning, attention, and working memory performance in healthy humans.

## Introduction

Noradrenaline (NA) is a major neuromodulator of the central nervous system (Bhagya et al., 2000; Robinson 2012). Via its extensive connections with multiple forebrain regions and the widespread distribution of noradrenergic receptors, the noradrenergic system is involved in arousal and response to acute stress and thought to modulate cognitive functions, including attention, memory, and learning (Bhagya et al., 2000; Robinson 2012). Noradrenergic activation alters neuronal excitability and regulates synaptic plasticity, which is thought to play a key role in cognitive performance at the neurophysiological level (Rioult-Pedotti et al., 2000; Balzarotti and Colombo, 2016). Cortical excitability and plasticity, including long-term potentiation (LTP) and long-term depression (LTD), are modulated by noradrenergic activation by its impact on various intracellular processes, including its effects on N-methyl-D-aspartate (NMDA) and on gamma-aminobutyric acid (GABA) receptors as well as on other neuromodulators, such as the dopaminergic system (Marzo et al., 2009). Animal studies have shown that neuronal excitability is enhanced by the activation of β-adrenoreceptors via suppressing GABAergic inhibition and facilitating the activation of NMDA receptors (Lei et al., 2007). On the other hand, α-adrenoreceptors decrease neural excitability by facilitating GABAergic inhibition, possibly via downregulation of calcium signaling. Similar results have been found in human studies (Marzo et al., 2009). Here, noradrenergic enhancement increases cortical excitability via enhancement of NMDA receptor–dependent facilitation and reduction of GABAergic inhibition in principle accordance with a primarily ß-adrenergic–enhancing effect (Wójtowicz et al., 2010; Kuo et al., 2017a).

Regarding synaptic plasticity, animal studies have shown that activation of β-adrenoreceptors strengthens LTP, while α-adrenoreceptors promote LTD (Linster et al., 2011; Bazzari and Parri, 2019). In a study conducted in humans, enhancement of monoamine availability fostered noninvasive brain stimulation–induced LTP-like plasticity, whereas stimulation-induced plasticity was reduced by a ß-adrenergic antagonist (Nitsche et al., 2004). Moreover, our foregoing study has shown that acute and chronic administration of the selective NA reuptake inhibitor reboxetine (RBX) increased and prolonged stimulation-induced LTP-like plasticity, whereas it converted LTD-like plasticity into LTP-like plasticity (Kuo et al., 2017b). Similar to adrenergic effects on excitability, this pattern of results is in accordance with a primary impact of ß-adrenoceptors on plasticity in humans.

Recent studies have shown that noradrenergic enhancement can increase synaptic plasticity as well as spatial learning in the animal model (Bhagya et al., 2015). Similarly, a single dose of RBX increased cortical excitability and improved the speed of motor task performance (rapid elbow flexion) in healthy participants (Plewnia et al., 2002; Plewnia et al., 2004). For depressed participants, noradrenergic enhancement increased memory and attention performance (Ferguson, et al., 2003; Chamberlain et al., 2006). Nevertheless, inconsistent results have also been reported (Kerr et al., 1996; Lange et al., 2007). The partially heterogeneous effects on cognitive performance might be explained by the complex effects of NA on brain physiology, dosage-dependent effects, and task characteristics. Importantly, there is strong evidence linking dysfunctions of the noradrenergic system to different neurological and psychiatric diseases, including depression, attention-deficit hyperactivity disorder, Parkinson’s disease, Alzheimer’s disease, and age-related decline of memory function (Ferguson et al., 2003; Marzo et al., 2009; Amano et al., 2013; Gannon et al., 2015). Taken together, the results of these studies suggest that the noradrenergic system significantly alters cortical excitability and plasticity in both animals and humans, which might be a foundation for the effects of NA on various cognitive functions.

These studies suggest that noradrenergic modulation of brain physiology might be an important foundation of cognitive performance. Generally, faster and more accurate performance might be due to enhanced cortical excitability, whereas learning and memory formation might be improved by plasticity enhancement in humans. Our previous studies have found that both acute and chronic RBX increased cortical excitability and LTP-like plasticity, which might be a relevant physiological mechanism for the functional effects of RBX. Here, we aimed to directly explore the impact of NA on cognitive functions in humans (Kuo et al., 2017a, 2017b). Specifically, we explored the impact of NA enhancement on motor sequence learning. We hypothesized that the NA-generated strengthening of LTP should result in improved learning and memory formation. Moreover, we explored the impact of NA enhancement on executive functions, namely working memory and attention. We hypothesized that the NA-dependent enhancement of glutamatergic and reduction of GABAergic activity will improve respective cognitive processes.

## Methods

### Participants

Twenty-five healthy right-handed and nonsmoking participants (12 females) aged 28.4 ± 3.02 (mean ± SD) years were recruited. None of them had a history of neurological or psychiatric diseases, pregnancy, or metallic head implants, nor did they take any medication during the study period. Written informed consent was obtained from all participants before inclusion. The investigation was approved by the Ethics Committee of the University Medical Center Goettingen and conforms to the Declaration of Helsinki.

### Pharmacological Intervention

Participants were asked to take RBX (8 mg) or equivalent placebo (PLC) drugs 2 hours before the start of each experimental session, allowing the verum to reach peak plasma level to produce prominent effects in the central nervous system (Pellizzoni et al., 1996; Dostert et al., 1997). The specific dosage was chosen because it elicited prominent effects in the central nervous system in previous studies (Plewnia et al., 2002; Ferguson et al., 2003; Kuo et al., 2017a).

### Serial Reaction Time Task (SRTT)

The SRTT is a standard paradigm to test implicit motor learning (Nissen and Bullemer, 1987; Exner et al., 2001). Participants were seated in front of a computer screen at eye level and a response pad placed on a table with 4 buttons numbered 1–4. They were instructed to push each button with a different finger of the right hand (index finger for button 1, middle finger for button 2, ring finger for button 3, and little finger for button 4). In each trial, an asterisk appeared in 1 of 4 positions that were horizontally spaced on a computer screen and permanently marked by dots. The participants were instructed to press the key corresponding to the position of the asterisks as fast and correctly as possible. After a button was pushed, the go signal (asterisk) disappeared. The next go signal was displayed 500 milliseconds after the participant pushed the button, without informing the participants about the correctness of the answer (Kuo et al., 2008). A test session consisted of 8 blocks of 120 trials each. In blocks 1 and 6, the sequence of asterisks followed a pseudo-random order. Asterisks were presented equally frequently in each position and never in the same position in 2 subsequent trials. In blocks 2 to 5 and in 7 and 8, the same 12-trial sequence of asterisk positions was repeated for 10 times (Nitsche et al., 2010). Two versions of sequences, which otherwise fulfilled the same criteria as the random stimulus order, were generated, and each participant received each version only in 1 session in counterbalanced order to avoid interference effects. Participants were not told about a repeating sequence.

### The Stroop Color-Word Test

The Stroop task is a neuropsychological test that measures cognitive flexibility, selective attention, cognitive inhibition, and information-processing speed (Bryan and Luszcz, 2000; Peña-Casanova et al., 2009; Grundey et al., 2015). The test includes 3 different sections in which the participant is asked to perform the task as quickly as possible. Stimuli were presented on a computer screen on a black background. The size of stimuli was 2.4 cm at approximately 50 cm eye distance. The first section is the Stroop word task. Here, participants were presented with 4 different words (red, blue, green, yellow) written in black ink. A keyboard with 4 keys, colored in red, blue, yellow, and green, was placed in front of the participants. The participants were asked to press the appropriate response key (word: green; green key, etc.) as fast and accurately as possible. The second section is the Stroop color. Here 4 Xs were presented in red, green, yellow, and blue ink, and again participants were asked to press the corresponding key on the keyboard as fast and accurately as possible. The third and last section is the Stroop color-word task (incongruent session). In this section, the color of the ink in which the word was written was different from the meaning of the word (e.g., the word “red” was written in blue). Participants had to press the corresponding key of the color in which the word was written. The inter-stimulus interval was 500 milliseconds. Participants were not informed if the task was performed correctly. Two versions of sequences were generated, and each participant received each version only in 1 session in counterbalanced order to avoid interference effects. The resulting increase in reaction time, compared with the other conditions, in the color-word condition is the color-word interference effect or Stroop effect. One section consisted of all 3 conditions with 15 trials each, resulting in 45 stimuli per section. This section was conducted 3 times.

### 3-Back Letter Task

We used the 3-back letter task to explore working memory performance. This task is sensitive to medication effects (Loughead et al., 2009; Grundey et al., 2015). Participants were seated in front of a computer monitor with 50-cm eye distance and presented with a pseudo-random set of 10 letters (A–J). Each letter was displayed on the computer monitor (14.1 in.) for 30 milliseconds. Black letters were presented on a white background and subtended 2.4 cm (when viewed at 50-cm eye distance). A new letter was displayed every 2 seconds. Participants were required to press a response pad (key press) only if the third-last stimulus was identical. Altogether 143 letters were presented, and a total of 30 correct responses were possible, depending on the version of the test. We applied different versions of the test to avoid learning effects. Before each session, participants were allowed to practice the task for 20 minutes or until they obtained an accuracy of 50% to exclude an impact of unspecific learning effects on performance. Participants were not informed about wrong and correct answers.

### Experimental Course

This experiment was performed in a cross-over and double-blind design, with randomized and counter-balanced order. Each participant took part in 2 experimental sessions (one PLC session and 1 RBX session). Two hours after RBX or PLC intake, the SRTT, STROOP test, and 3-back letter task were conducted in randomized order. These tasks were selected due to their sensitivity to medication effects, their wide application in the previous literature, and because they cover different domains of cognitive functions, which are thought to be affected by NA (Ferguson et al., 2003; Chamberlain et al., 2006; Loughead et al., 2009). Participants were allowed to take breaks between each cognitive task. The duration of the respective breaks was chosen freely by the participants to enable an appropriate break to keep attention constant. Each psychological test required 15 minutes in average, and about 1 hour was needed to finish all tests. To avoid medication or task interference effects, a 1-week break between sessions was obligatory.

### Data Analysis

For the SRTT, in each trial, response time (RT) was recorded from the appearance of the go signal until the first button was pushed by the participant. For each block of trials of a given experimental session, mean RT was calculated for each participant separately. Incorrect responses, RTs of <200 ms, >3000 ms, or those that were above 3 SDs of the individual mean RT were discarded. Mean RT were standardized to block 1 for each participant in each medication condition separately to control for initial RT differences. Furthermore, the SD of RT for each participant in every block was calculated as an index of variability. Error rate (ER) was calculated to assess the number of incorrect responses for each block and each participant in each session. Statistical analysis was performed for the absolute and standardized values of RT, ER; variability of RT via repeated-measures ANOVA (level of significance = .05); the within- participant factors medication (PLC vs RBX); and block. The Mauchly test was performed to test for sphericity, and the Greenhouse-Geisser correction applied when necessary for these and the following ANOVAs. Dependent on significant results in the ANOVAs, RT, ER, and variability value differences between the respective medication conditions were compared by paired samples 2-tailed Student’s *t* tests (level of significance = .05) for each block of the task and between blocks for a given medication condition. Because RT differences between block 5 and 6 are thought to represent an exclusive measure of implicit sequence motor learning, interactive Student’s *t* tests were conducted to compare differences of respective RTs between these intervention conditions (blocks 5 and 6 of the SRTT under PLC and RBX).

In the Stroop color-word test, the respective individual means of the corresponding reaction times, percentage (percentage of right answers), and errors were calculated for each session for the conditions word, color, and incongruent. Repeated-measures ANOVAs were conducted for the respective dependent variables. Within-participant factors were drug (PLC vs RBX) and sequence (word, color, incongruent). The Mauchly test was performed to test for sphericity and the Greenhouse-Geisser correction applied when necessary for these and the following ANOVAs. Conditional on significant results of the ANOVA, paired-sample 2-tailed *t* tests (comparing participants under PLC or RBX) were performed for post hoc analysis.

For the 3-back letter task, the primary outcomes were hits, misses, correct rejections, false alarms, and reaction time. Furthermore, the sensitivity index d’ was calculated for both conditions (Haatveit et al., 2010). The index d’ is derived from signal detection theory and reflects the ability to discriminate targets from nontargets. d’ was calculated with the following formula: Z (hit rate) − Z (false alarm rate), where Z represents the z-scores of both rates (Macmillan and Creelman, 1991). Perfect scores were adjusted using these formulas: 1-1/ (2n) for perfect (e.g., hit rate) and 1/(2n) for zero false alarms. For each participant, an individual mean was calculated for each of these variables. Paired-sample 2-tailed tests were applied to compare outcomes under RBX and PLC for the respective variables. A *P* value < .05 was considered significant for all statistical analysis. Exploratory post hoc tests were not corrected for multiple comparisons. All data are expressed as mean ± SEM. Analysis were performed with IBM SPSS Statistics Version 22.

## Results

All participants completed the entire study. Only 2 participants complained about mild dizziness (under the RBX condition), which was well controlled by taking a rest for 30 minutes.

### SRTT

**Figure 1.**
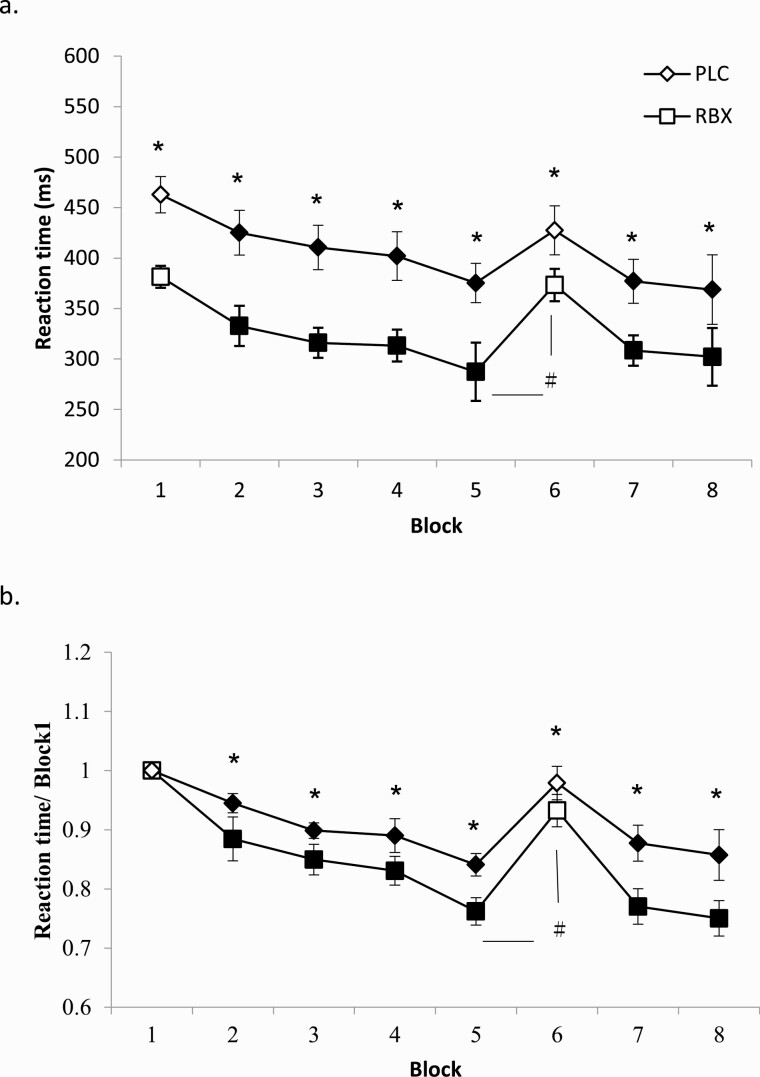
Serial reaction time task (SRTT) performance (reaction time). Depicted are the (A) mean absolute reaction time (ms) and (B) standardized reaction time for each intervention condition (blocks 1–8). In blocks 1 and 6, random stimuli were presented and in the remaining blocks, the sequence was presented. The results show that participants became faster during learning in both PLC and RBX conditions. In addition, reaction time was generally significantly shorter in the RBX condition over all blocks. For both A and B, the reaction time difference between block 5 and 6, which is a pure index of motor learning, was larger for the RBX compared with the PLC condition, indicating improved learning under RBX. Filled symbols indicate significant reaction time differences of RBX/PLC conditions relative to the respective block 1, and the asterisks indicate significant differences between PLC/RBX conditions for a single block (2-tailed *t* tests, paired samples, *P* < .05). Hash symbols indicate a significant difference of the RT difference between block 5 and 6 with respect to the RBX/PLC condition (2-tailed, *t* test, paired samples, *P* < .05). Error bars in this and the following figures represent the SEM.

As displayed in Table 1, for absolute RT, the repeated-measures ANOVA revealed significant main effects for the factor block (F[7] = 18.286; *P* < .001), drug (F[1] = 37.409; *P* < .001), and drug × block interaction (F[7] = 3.161; *P* = .021). For standardized RT, significant main effects for the factor block (F[7] = 17.705; *P* < .001) and drug (F[1] =  29.714; *P* < .001) emerged. For absolute RT, the main effect of block is caused by reduced RTs compared with random block 1 in the later sequences (Fig. 1), with the exception of the random block 6, which did not contain the learned sequence. The main effect of the factor drug was caused by the fact that for the RBX condition, RTs were significantly smaller compared with the PLC condition for all blocks (block 1: *t* value 7.759, *P* < .001; block 2: *t* value 5.986, *P* < .001; block 3: *t* value 6.319, *P* < .001; block 4 *t* value 5.064, *P* < .001; block 5: *t* value 2.784, *P* = .011; block 6: *t* value 2.966, *P* = .001; block 7: *t* value 2.996, *P* = .007; block 8: *t* value 5.886, *P* < .001) as shown in Figure 1A. Similar results were obtained for standardized RT. Standardized RTs were significantly smaller under RBX compared with the PLC condition for most of the blocks (block 2: *t* value 1.856, *P* = .046; block 3: *t* value 1.916, *P* = .038; block 4: *t* value 1.841, *P* = .049; block 5: *t* value 2.435, *P* = .023; block 6: *t* value 2.090 *P* = .038; block 7: *t* value: 2.086, *P* = .038; block 8: *t* value 3.153, *P* = .004) (Fig. 1B). With respect to the significant interaction, as revealed by the post hoc *t* tests, the difference in absolute and standardized RT between block 6 and 5 (RT6 − RT5) under RBX was significantly larger than that under PLC, reflecting learning in both conditions but improved learning under RBX (absolute RT: *t* value 3.987, *P* = .001; standardized RT: *t* value 3.103, *P* = .004). For ER and variability, the repeated-measures ANOVAs show no significant main effects of drug, block, or the respective interactions (all *P* > .05).

**Table 1.** Repeated-Measures ANOVAs Performed for the SRTT and STROOP Color-Word Test

Test	Parameters	Conditions	df	F value	*P* value
SRTT	RT (absolute)	Block	7	18.286	**<.001** ^***a***^
		Medication	1	37.409	**<.001** ^***a***^
		Medication × block	7	3.161	**.021**
	RT (standardized)	Block	7	17.705	**<.001** ^***a***^
		Medication	1	29.714	**<.001** ^***a***^
		Medication × block	7	1.823	.082
	Variability of RT	Block	7	2.505	.18
		Medication	1	0.732	.425
		Medication × block	7	0.93	.494
	Errors	Block	7	6.063	.748
		Medication	1	0.113	.748
		Medication × block	7	1.168	.342
Stroop	Reaction time	Sequence	2	5.532	**.039** ^***a***^
		Medication	1	5.653	**.049** ^***a***^
		Medication × sequence	2	0.022	.925
	Errors	Sequence	2	15.613	**<.001** ^***a***^
		Medication	1	2.896	.102
		Medication × sequence	2	3.118	**.045** ^***a***^
	Percentage	Sequence	2	1.011	.377
		Medication	1	1.004	.333
		Medication × sequence	2	1	.381

Abbreviations: df, degrees of freedom; RT, reaction time.

^*a*^The bold font indicates significant results at *P* < .05.

### Stroop Task

**Figure 2.**
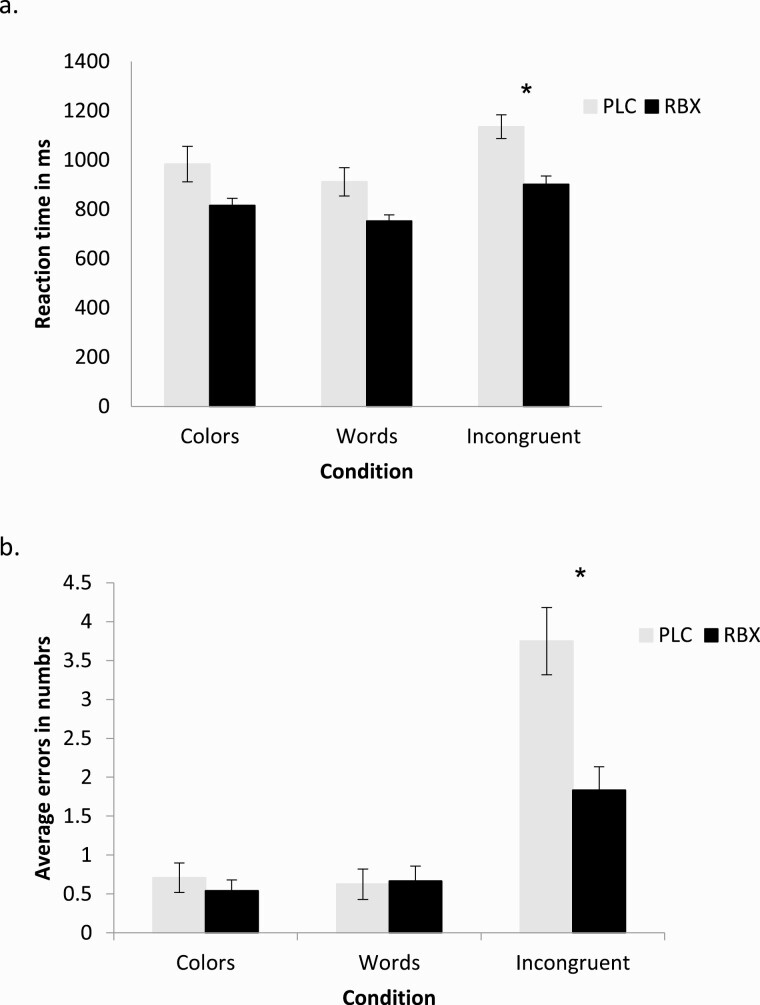
Results of the Stroop test under RBX and PLC conditions for reaction time in milliseconds (A) and averaged number of errors (B). Compared with the PLC condition, administration of RBX improves performance in terms of reaction time and number of errors in the incongruent condition. Asterisks represent significant differences between PLC and RBX conditions (2-tailed, *t* tests, paired samples, *P* < .05). Vertical bars depict the SEM.

Regarding reaction time (see also Table 1), the repeated-measures ANOVA revealed significant main effects of condition (F[2] = 5.532; *P* = .039) and drug (F[1] = 5.653; *P* = .049). The post hoc *t* tests (paired-sample *t* tests) show that under RBX, participants were significantly faster compared with the PLC condition in the color-word incongruent condition (*t* value 5.877, *P* < .001) (Fig. 2). For errors, the repeated-measures ANOVA yielded significant results for the main effect condition (F[2] = 15.613; *P* < .001) and the interaction between drug × condition (F[2] = 3.118; *P* = .045). The post hoc *t* tests show that RBX significantly decreased the number of errors in the color-word incongruent condition (*t* value −5.007, *P* < .001) (Fig. 2).

### 3-Back Letter Task

**Figure 3.**
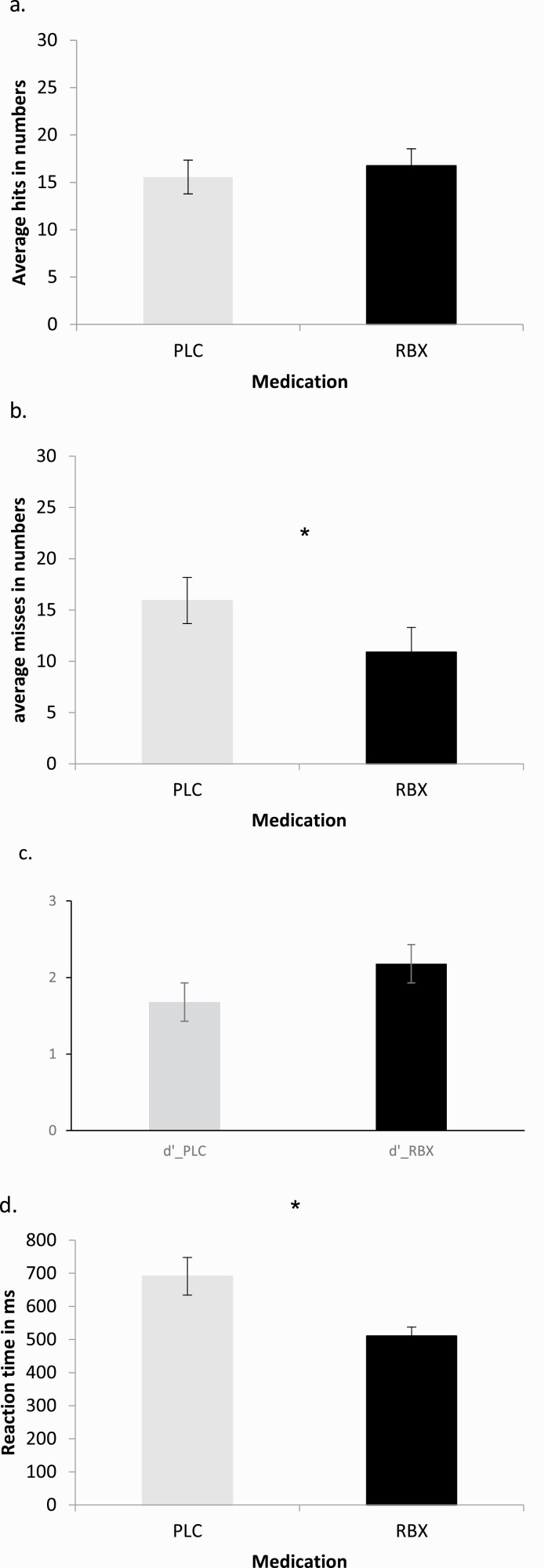
The results of the 3-back letter task for (A) averaged hits, (B) averaged misses, (C) d’, and (D) reaction time after administration of RBX or PLC. RBX shortened reaction times and reduced misses compared with the PLC condition. Asterisks represent significant differences between PLC and RBX (2-tailed, *t* tests, paired samples, *P* < .05). Vertical bars depict the SEM.

The paired-samples *t* test revealed a significant effect of reaction time (reduced reaction time) (*t* value 6.333, *P* < .001) and misses (reduced misses) (*t* value 2.922, *P* = .008). Hits, correct rejections, false alarms, and d’ did not differ between intervention conditions (all >0.05) (Fig. 3). Under RBX, participants thus improved significantly in terms of reaction time and misses but not in other performance parameters (Table 2).

**Table 2:** Results of the Paired *t* Tests Conducted for the 3-Back Letter Task

	*t* value	*P*
Reaction time	6.333	**<.001** ^*a*^
Hits	1.088	.318
Misses	2.922	**.008**
Correct rejections	0.773	.469
False alarms	0.027	.979
Index d’	0.877	.409

^*a*^The bold font highlights significant results at *P* < .05.

## Discussion

The results of this study show that at the dosage applied in the present experiments, RBX improved various cognitive functions, including learning, and executive functions. Specifically, RBX enhanced motor sequence learning, as shown by SRTT, and flexibility/selective attention as shown by the STROOP task results. It furthermore improved reaction time and reduced the number of misses in the working memory task.

For the SRTT, the results of our study show that RT was significantly shortened in all blocks under RBX compared with the PLC condition. This shows that RBX improved performance independently from sequence learning. More importantly, however, RBX additionally improved performance in the sequence block when the impact of the learning-unspecific RT improvement was excluded and thus enhanced sequence learning performance. This result is in accordance with related animal experiments, where it was shown that RBX has a beneficial effect on 5-choice SRTT performance in rats (Robinson, 2012). In contrast, ER and variability were not modified by the intervention. Thus, the RT effects reflect true performance improvements, which were not achieved on the cost of error or variability enhancement. These effects might be caused by the excitability-enhancing effect of RBX, which might bring task-activated synaptic connections nearer to their synaptic modification threshold, thus facilitating LTP. More specifically, this process might be driven by NMDA receptor activity enhancement and gated by GABA activity reduction, both induced by RBX, which are relevant for motor learning and memory formation (Hasan et al., 2013; Kolasinski et al., 2019). Indeed, animal studies have shown that NA enhancement induces glutamatergic and calcium-dependent LTP (Maity et al., 2015; Jȩdrzejewska-Szmek et al., 2017). This is furthermore in accordance with previous studies from our and other groups, which describe enhanced cortical excitability and LTP-like plasticity after application of RBX in humans (Plewnia et al., 2002; Kuo et al., 2017b). Therefore, RBX-driven cortical excitability and plasticity enhancement might be an important neurophysiological foundation for the improvement of motor learning observed in this experiment. Nevertheless, a former study describes no effect of RBX on motor skill acquisition (finger tapping) (Lange et al., 2007). This deviating effect is, however, most likely explained by the relevantly lower dosage of RBX (2 mg) applied in that study.

For Stroop task performance, the results revealed a positive overall effect on reaction time under RBX but—similar to the SRTT results—an additional specific effect on selective attention. Our findings are in line with results of previous studies showing that blockade of NA receptors impairs selective attention in animal models (Ma et al., 2003, 2005). Similarly, in studies conducted in humans, RBX improved attention in major depression (Ferguson et al., 2003). Probable mechanisms of action include NMDA receptor activity enhancement as accomplished by RBX. Stroop task performance has been shown to be prominently affected by glutamatergic activation (Stoet and Snyder 2006; Kühn et al., 2016). These results imply, moreover, that RBX works most prominently in demanding attentional processes, which might require a larger amount of glutamatergic activation (Adleman et al., 2002). Alternatively, it cannot be ruled out that for the easier task conditions, only a somewhat smaller effect of RBX emerged in the present study because of a ceiling effect.

For working memory performance, noradrenergic activation improves working memory in rats and monkeys (Birnbaum et al., 2000; Ramos and Arnsten 2007). In accordance, human studies have found that a single dose of RBX improved working memory in healthy and depressed participants (Ferguson et al., 2003; Chamberlain et al., 2006). However, 1 study found no effects on RBX applied in relatively low dosages (0.5/1/4 mg) on Sternberg test performance (recognition probe for previously memorized digit sequences) in healthy participants (Kerr et al., 1996). These partially inconsistent effects between studies might thus be caused by different dosages of RBX and task characteristics. In the present study, participants showed improved working memory performance (reduced reaction time and number of misses) under RBX. NMDA-Rs and the glutamate system are critically involved in working memory performance (Driesen et al., 2013). RBX enhances glutamatergic activity. In healthy humans, it was shown that RBX increases intracortical facilitation, which is known to primarily controlled by the glutamatergic system (Kuo et al., 2017a). These neurophysiological mechanisms of RBX thus provide a plausible explanation for its enhancing effects on working memory performance.

Beneath RBX, other substances have also been shown to have cognition-enhancing effects in healthy humans, although not always to the same extent. Regarding indirect agonists, caffeine has been shown to increase the accuracy but not reaction time of working memory task performance (3-back letter task), whereas it had no effects on STROOP task performance in healthy participants (Edwards et al., 1996; Ueda and Nakao 2019). Furthermore, psychostimulants such as modafinil and methylphenidate improved RT and ERs of STROOP task performance in the incongruent condition but had no significant effects on choice reaction time and 3-back letter task performance in healthy participants (Minzenberg and Carter 2008; Campbell-Meiklejohn et al., 2012; Wood et al., 2014; Turner et al., 2019). However, due to experimental differences, it is difficult to compare directly the magnitude of effects achieved by RBX, caffeine, and the respective psychostimulants. Moreover, no studies are available that compared directly different substances with respect to identical tasks in the same participant groups, which would be ideal for such a comparison. One explanation for the performance-improving effect of noradrenergic enhancement in our participants, which is not seen for all cognitive enhancers, is that young healthy participants do not reach their maximum possible performance level in each case under normal conditions. There is a well-known performance reserve, which is stress related, and can improve performance further (Robertson, 2013; Cabral et al., 2016). Activation of this performance reserve is associated with NA enhancement (Robertson, 2013).

Some limitations of the present study should be taken into account. First of all, the cognitive performance measurements and related neurophysiological studies conducted by our group and others in previous experiments were conducted in different groups of participants. Thus, statements about causal relations of respective effects are speculative at present. However, similar demographic characteristics of the groups explored in the physiological and cognitive studies conducted by our group allow preliminary conclusions to be drawn. Secondly, the results of our study partly differ from those of former cognitive studies with noradrenergic agents. Inconsistent results might be based on different noradrenergic receptor activation, medication dosages, and task characteristics. There is increasing evidence that neuromodulators may operate nonlinearly, such as dopamine, where under- or overactivity impairs cognitive processes, while medium activity leads to optimized performance (Cools and D’Esposito, 2011; Monte-Silva et al., 2011; Fresnoza et al., 2014). To our knowledge, no studies so far systematically explored the nonlinear effects of NA, but for amphetamine, which has NA-activating effects, respective nonlinearities have been described. Nevertheless, specific titration studies might be required. Furthermore, we did not ask the participants if they thought they received real or PLC medication after the end of the experiment. Because previous studies described that participants cannot distinguish between PLC and real medication with 8 mg RBX (Wang et al., 2009), we assumed that the double-blind design was reliable. Direct exploration of the integrity of blinding might, however, be advantageous in future studies. Finally, the present study was conducted in healthy participants. In neuropsychiatric diseases, transmitter availability and other features of brain functions might be different. Future studies are thus needed to explore the transferability of these results to patients. Some technical limitations of our study are that we did not obtain plasma levels of RBX and did not conduct peripheral measures of drug effects, such as blood pressure and pulse frequency, which might have further helped to establish respective dose-effect relationships.

## Conclusion

In the present study, we examined the impact of noradrenergic enhancement with the NA reuptake inhibitor RBX on cognitive performance with regard to learning and memory formation, and executive functions, namely attention and working memory performance. Beyond a general unspecific beneficial effect on reaction time, the results provide evidence for a prominent involvement of noradrenergic system activation in implicit motor learning, attention, and working memory performance in healthy humans. When associated with the results of related neurophysiological studies, these cognitive effects are most likely caused by respective NA-related effects on cortical plasticity and excitability. The results of these studies provide a potential mechanism to explain the improvement of daily functioning observed in patients treated by noradrenergic agents and might also offer opportunities to exploit respective agents to counteract cognitive decline or improve rehabilitation results.

## Statement of Interest

H.-I.K., F.-X.Q., and M.-F.K. received no ﬁnancial support or compensation from any individual or corporate entity over the past 3 years for research or professional service, and there are no personal ﬁnancial holdings that could be perceived as constituting a potential conﬂict of interest. W.P. is a member of the advisory boards of GSK, UCB, and Desitin. M.A.N. is a member of the advisory boards of Neuroelectrics and NeuroDevice. All authors declare no financial and personal relationships with other people or organizations that can inappropriately influence the work. There is no professional interest of any nature or kind in any product, service and/or company that could be constructed as influencing the position presented in, or he manuscript entitled.
